# Bacterial Biofilm in Chronic Wounds and Possible Therapeutic Approaches

**DOI:** 10.3390/biology13020109

**Published:** 2024-02-09

**Authors:** Ilaria Cavallo, Francesca Sivori, Arianna Mastrofrancesco, Elva Abril, Martina Pontone, Enea Gino Di Domenico, Fulvia Pimpinelli

**Affiliations:** 1 Microbiology and Virology Unit, San Gallicano Dermatological Institute IRCSS, 00144 Rome, Italy; ilaria.cavallo@ifo.it (I.C.); francesca.sivori@ifo.it (F.S.); elva.abril@ifo.it (E.A.); martina.pontone@ifo.it (M.P.); fulvia.pimpinelli@ifo.it (F.P.); 2 Department of Biology and Biotechnology “C. Darwin”, Sapienza University of Rome, 00185 Rome, Italy; enea.didomenico@uniroma1.it

**Keywords:** wound healing, chronic wounds, skin microbiota, chronic wound infections, biofilm, anti-biofilm therapeutic approaches

## Abstract

**Simple Summary:**

Chronic wounds consist of those injuries that have failed to complete the healing process. This type of wound is frequently infected by pathogens and represents a challenging medical situation, a substantial cause of health problems, and a financial burden for the healthcare system. Indeed, the ability of some pathogens to produce multicellular structures enclosed in a matrix, called biofilms, considerably hampers the efficacy of the treatments. Hence, this work aims to strengthen the knowledge of the pathophysiology and treatment of infected chronic wounds. With this purpose, this work gives a comprehensive overview of the wound healing process, the pathogenesis of chronic wounds, with a special focus on chronic wounds infected by biofilm-forming pathogens, and on anti-biofilm therapeutic strategies. The strategies currently used in the clinical setting to remove biofilms from chronic wounds are described alongside several approaches currently under development. These novel strategies have the potential to counteract the ability of pathogens to produce biofilms, kill the pathogens within the biofilm, target biofilm molecules, or activate the immune system against the infection. These strategies used in combination could result in the better management of patients, avoiding the development of serious healthcare outcomes.

**Abstract:**

Wound repair and skin regeneration is a very complex orchestrated process that is generally composed of four phases: hemostasis, inflammation, proliferation, and remodeling. Each phase involves the activation of different cells and the production of various cytokines, chemokines, and other inflammatory mediators affecting the immune response. The microbial skin composition plays an important role in wound healing. Indeed, skin commensals are essential in the maintenance of the epidermal barrier function, regulation of the host immune response, and protection from invading pathogenic microorganisms. Chronic wounds are common and are considered a major public health problem due to their difficult-to-treat features and their frequent association with challenging chronic infections. These infections can be very tough to manage due to the ability of some bacteria to produce multicellular structures encapsulated into a matrix called biofilms. The bacterial species contained in the biofilm are often different, as is their capability to influence the healing of chronic wounds. Biofilms are, in fact, often tolerant and resistant to antibiotics and antiseptics, leading to the failure of treatment. For these reasons, biofilms impede appropriate treatment and, consequently, prolong the wound healing period. Hence, there is an urgent necessity to deepen the knowledge of the pathophysiology of delayed wound healing and to develop more effective therapeutic approaches able to restore tissue damage. This work covers the wound-healing process and the pathogenesis of chronic wounds infected by biofilm-forming pathogens. An overview of the strategies to counteract biofilm formation or to destroy existing biofilms is also provided.

## 1. Introduction

The skin is a complex organ that is essential for survival since it is responsible for the protection of the internal organs from pathogen colonization and the maintenance of a state of hydration. The skin structure consists of three differentiated layers, each with specific features and cell composition: the epidermis, the dermis, and the hypodermis [1]. The epidermis is the upper layer composed principally of keratinocytes that play essential roles in skin repair. The middle layer, the dermis, consists mainly of an extracellular matrix (ECM) and provides the majority of structural and mechanical strength to the skin, while the hypodermis is the innermost skin layer, mostly consisting of adipose tissue that displays a connective function between the skin and either muscles or bones [2,3]. The ability of the skin to continuously self-renew and to heal from damage is essential in maintaining homeostasis [4]. Specifically, a skin wound is damage caused by laceration, and the process of repair, wound healing, is essential to restore the normal barrier function of the skin, preventing infection. Wound healing is a highly regulated and dynamic process that involves the participation of various immune and structural cells and follows four phases: hemostasis, inflammation, proliferation, and remodeling [4,5]. Failure to proceed through the phases of the healing process in an orderly and timely manner leads to chronic wounds [6]. Chronic wounds represent a major health concern, and their management is particularly challenging, especially in the presence of infections [7].

In this review, we present the wound-healing process and the characteristics of chronic wounds, with a special focus on infected chronic wounds. We also present the roles of bacterial biofilms and the skin microbiota, respectively, in delaying and promoting wound healing. Finally, we present strategies that, by reducing biofilms in chronically infected wounds, can promote the resolution of infections and wound healing. 

## 2. The Normal Wound Healing Process

### 2.1. Hemostasis

Immediately after an injury, the hemostatic process takes place in order to stop bleeding and produce a provisional matrix that the ECM deposited by fibroblasts will subsequently substitute. In this process, platelets become activated by interaction with exposed collagen and start to release growth factors, cyclic AMP, and chemokines, triggering cellular signaling that confers them with the ability to aggregate [8]. Afterward, fibrin deposition occurs, and platelets become trapped in this matrix, forming a clot [9]. Platelets produce several factors, including transforming growth factor β (TGF-β), TGF-α, platelet-derived growth factor (PDGF), vascular endothelial growth factor (VEGF), basic fibroblast growth factor (bFGF), and insulin growth factor (IGF). These factors, besides recruiting monocytes and neutrophils to start the inflammatory phase, promote angiogenesis and recruit fibroblasts that start ECM production. Once recruited, monocytes will differentiate into inflammatory M1 macrophages [10]. The essential role that platelets play in wound repair is widely exploited at the clinical level through the use of platelet-rich plasma (PRP) to promote healing [11].

### 2.2. The Inflammatory Phase

Epidermal keratinocytes, neutrophils, and macrophages are the main cell types involved in the inflammatory response. As the first line of defense, keratinocytes play a critical role in wound repair not only as structural cells but also exert important immune functions. Through pattern-recognition receptors (PRRs), keratinocytes recognize several pathogen-associated molecular patterns (PAMs) leading to the activation of distinct signaling pathways and the production of inflammatory cytokines, chemokines, and host antimicrobial molecules [12]. All these mediators play a crucial role in activating skin-resident immune cells and recruiting circulating immune cells to the wound site [13]. Skin-resident immune cell populations include αβ and γδ T-cell receptor (TCR)-expressing T cells and are critical in orchestrating key aspects of wound healing. In particular, epidermal and dermal γδ T cells promote complex crosstalk with keratinocytes and inflammatory cells to maintain skin homeostasis [14]. Neutrophils play a central role in healing damaged tissue and resolving infections. In fact, they are the first immune cells to arrive at the damaged tissue and produce cytokines, such as interleukin (IL) 1β (IL-1β), IL-6, and tumor necrosis factor α (TNF-α), and chemokines, e.g., CXCL2, CXCL8, able to attract other immune cells amplifying the inflammatory response. Moreover, neutrophils release H_2_O_2,_ leading to the production of reactive oxygen species (ROS) capable of eliminating debris and bacteria, promoting antimicrobial protection [15]. ROS not only protect the wound from infection but also stimulate the multiplication of fibroblasts, the proliferation and migration of keratinocytes and angiogenesis [16]. In addition, ROS, together with gradients of growth factors, proinflammatory cytokines, and chemokines produced by hyperproliferative keratinocytes, are responsible for macrophage recruitment at the wound edge. At this stage, macrophages show a classically activated M1-like phenotype producing inflammatory mediators, such as IL-1β, TNF-α, IL-6, IL-12, and IL-23, able to attract additional immune cell recruitment, thus amplifying the inflammatory response [6]. Moreover, they clear debris and invading microorganisms and eliminate neutrophils [17], thus avoiding ROS overproduction that would induce states of chronic inflammation that lead to skin lesions [18]. Subsequently, macrophages acquire an anti-inflammatory M2-like phenotype characterized by markers of inflammatory resolution. Macrophages also secrete cytokines, pro-angiogenic factors, and fibrogenic factors that attract cells for the proliferation phase of wound healing. 

### 2.3. The Proliferation Phase

During the proliferation phase, M2 macrophages release growth factors, such as VEGF, platelet-derived growth factor (PDGF), and FGF-2, able to promote angiogenesis and keratinocyte and fibroblast proliferation [17,19]. In this scenario, keratinocytes are the executors of the re-epithelialization process since they migrate at the wound edges where they proliferate and differentiate to establish coverage of the wound site, thus restoring the epidermal barrier [19,20]. In addition, stem cells from the bulge of the hair follicle or at the base of the sebaceous gland contribute to the wound re-epithelialization process [21]. The contribution of local adipocyte and melanocyte progenitor cells in the wound repair process has also been demonstrated [22]. In particular, adipose-derived stem cells (ADSCs) stimulate cell re-epithelialization and angiogenesis and regulate macrophages and cellular immunity [23]. This phase is also characterized by the deposition of the ECM by fibroblasts, leading to the formation of the granulation tissue that consists of a dense population of cells enclosed in a loose ECM composed of collagen, fibronectin, and hyaluronic acid [24].

### 2.4. The Remodeling Phase

Remodeling is the last phase of wound healing that leads to the regeneration of the skin barrier [25]. Fibroblasts are the major players in the remodeling phase. A balance between the production and disintegration of ECM components, such as collagen, elastin, and glycosaminoglycans (GAGs), is essential [26]. Dermal fibroblasts are also involved in the wound-healing process through their proliferation, migration, and ability to respond to cytokines. TGF-β is essential for fibroblasts to differentiate into myofibroblasts that display contractile activity due to the expression of alpha-smooth muscle actin [27]. During the remodeling phase, myofibroblasts promote the active contraction of the granulation tissue. In this phase, ECM components are modified to form a stronger and more organized matrix [28]. Hence, the normal healing process, by providing deposition and reorganization of the ECM, leads to scar formation. Importantly, the failure of the maturation and modeling phase should be due to the accumulation of excessive collagen and the formation of hypertrophic scars and keloid scars with strong rigidity. The mechanism of the formation of hypertrophic scars and keloids has not yet been completely understood, however, and the persistence of myofibroblasts in closed wounds has been observed [29]. In addition, many studies reported that excessive inflammation might lead to chronic and destructive pathological scarring. The interruption or deregulation of one or more phases of the wound-healing process leads to chronic wounds [30].

**Figure 1:**
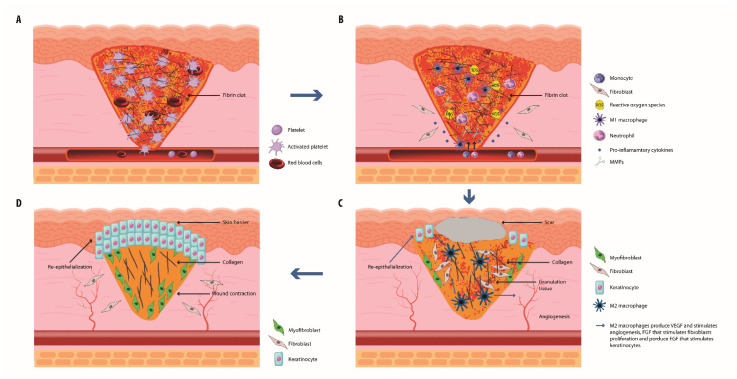
**Stages of wound healing.** (**A**) The process starts when platelets become activated by exposed collagen, start to aggregate, and become trapped in fibrin fibers, forming a clot. (**B**) Afterwards, the inflammatory phase starts with neutrophil recruitment that, by producing ROS, avoids wound infection and promotes fibroblast proliferation and angiogenesis. Subsequently, monocytes are recruited and differentiate into M1 macrophages that produce proinflammatory cytokines and clear neutrophilic debris. (**C**) During the proliferation phase, macrophages differentiate into M2 macrophages that produce EGF that stimulates keratinocytes to start the re-epithelialization, VEGF that promotes the angiogenesis process, and FGF that stimulates the multiplication of fibroblasts that start to deposit collagen replacing the fibrin clot with granulation tissue. (**D**) Finally, ECM produced by fibroblasts replaces the granulation tissue, the skin barrier is restored, and thanks to the activity of myofibroblasts, the wound contracts.

The phases of wound healing are summarized in Figure 1.

## 3. Commensal Skin Microbiota and Its Role in Wound Healing

Humans live in cooperation with their microbiota, defined as a community of bacteria, fungi, and viruses that inhabit different parts of the body, such as the skin, gastrointestinal tract, conjunctiva, oral cavity, vagina, uterus, and lungs [31,32,33]. Culture-based and metagenomics approaches demonstrated that the human skin microbiota comprises about 10^12^ bacteria, fungi, and viruses [34]. The most prevalent bacteria belong to the Actinobacteria, Firmicutes, and Proteobacteria phyla, while *Malassezia* species predominate among fungal communities. If, on one side, the skin microbiota must resist the skin’s hypersaline and acidic environment and the absence of several nutrients, on the other side, the skin offers protective niches and nutrients for microbial survival, competition, and cooperation. While moist areas of the skin are enriched by *Corynebacterium* species, *Cutibacterium acnes*, which uses sebum as a nutrient, dominates the hypoxic niche constituted by the pilosebaceous unit. Other signature bacteria of the skin microbiota comprise coagulase-negative staphylococci (CoNS) species, such as *Staphylococcus epidermidis*. The skin microbiota enhances the skin barrier by taking part in the defense of the body by directly interacting with commensal and pathogenic microbes and modulating the immune system [35]. In polymicrobial communities, skin resident microorganisms have evolved antagonism mechanisms to inhibit the growth of other species. For example, *Staphylococcus hominis* is able to produce antibiotic molecules to inhibit the growth of *Staphylococcus aureus* [36], while *Staphylococcus capitis* antagonizes *S. aureus* through interference with the gene regulator (*agr*), required for its virulence [4,37]. Moreover, some skin microbiota species bolster skin immunity by stimulating the production of host-derived antimicrobial peptides (AMPs), which kill bacteria by creating pores on their membranes [38]. A crosstalk between the host and the microbes guarantees the maintenance of the skin microbiota through the establishment of mechanisms of immunological tolerance through the coordinated activity between keratinocytes, which function as non-professional antigen-presenting cells (APCs), and regulatory T cells. In this way, the immune system discriminates the resident skin microorganisms from pathogenic ones without activating the inflammatory response but promoting commensal tolerance [39]. The skin microbiota takes part during wound healing, coordinating the innate immune response. Indeed, studies have also shown that the accumulation of Tregs specialized in guaranteeing tolerance toward commensal microorganisms promotes healing by allowing the colonization of wounds by those beneficial microorganisms [40]. It has been demonstrated that the skin microbiota activates plasmacytoid dendritic cells, which generate the production of type I interferon on the site of the injury, accelerating wound repair through stimulation of fibroblast and macrophage growth factor responses [41]. Moreover, the skin commensal *S. epidermidis* contributes to the skin barrier integrity by supporting ceramide production, the principal lipid component of the skin barrier [42]. It is, therefore, clear that commensal microorganisms display an essential function in inhibiting the growth of pathogenic species, thus maintaining homeostasis, as well as promoting wound healing. 

## 4. The Impaired Healing Resulting in Chronic Wound

Chronic wounds are defined as those injuries that have failed to proceed through the phases of the healing process in an orderly and timely manner. Chronic wounds are classified into different categories, such as diabetic foot ulcers (DFUs), venous leg ulcers (VLUs), pressure ulcers (PUs), surgical site infections (SSIs), abscesses, or trauma ulcers [6]. Several factors could be responsible for the delay or failure of healing of chronic wounds, including the patient’s age, nutritional state, the presence of diabetes, or immunocompromised condition (e.g., cancer). Moreover, some therapies, such as chemotherapeutic agents, radiotherapy, and long-term use of corticosteroids and non-steroidal anti-inflammatory drugs, could interfere with wound healing [43]. Chronic wounds are susceptible to infections that can lead to serious health problems, such as amputation and even death [44,45]. Hence, detailed knowledge of chronic wounds is required in order to develop better wound treatment and management strategies. In contrast to the normal process of wound healing, angiogenesis, stem cell recruitment and activation, and ECM remodeling have all been shown to be impaired in chronic wounds, whereas the inflammatory state has been seen as persistent and unresolved [19]. In fact, the common features of non-healing wounds are exudation, repeated infection, tissue necrosis, defective re-epithelization, decreased angiogenesis, and overproduction of ROS [17,46]. Chronic inflammation is a well-known hallmark of chronic wounds and is due to a deregulation of the immune response with an increase of proinflammatory infiltrates composed of neutrophils and macrophages that contribute to delayed healing in chronic ulcers. The deregulation of several key proinflammatory cytokines, such as IL-1β and TNF-α, maintains an inflammatory phase, which causes a delay in healing [47]. Excessive degradation and damage of the ECM are caused by the increased levels of metalloproteinases (MMPs) triggered by IL-1β and TNF-α [48] and by the overproduction of ROS by neutrophils [49]. The exaggerated ECM destruction perpetuates the inflammatory response and fuels pathogens to form multicellular structures called biofilms, which represent a hallmark of chronically infected wounds [50]. In addition, the absence of the skin resident γδ T cells has been associated with impaired healing because these cells are involved in crosstalk with keratinocytes that coordinate re-epithelization and inflammation [14]. The resulting changes in ECM degradation and deposition, immune system aberration, and the compromised angiogenesis process promote stagnation into the inflammatory phase, thus leading to chronic wounds. The highly inflammatory and hypoxic environment also leads to tissue necrosis [46]. The development of chronic non-healing wounds is triggered by many factors, including reduced microcirculation, decreased availability of cytokines and growth factors that promote wound closure and healing, skin dysbiosis, and infections by multi-drug resistant microbes [51]. 

**Figure 2:**
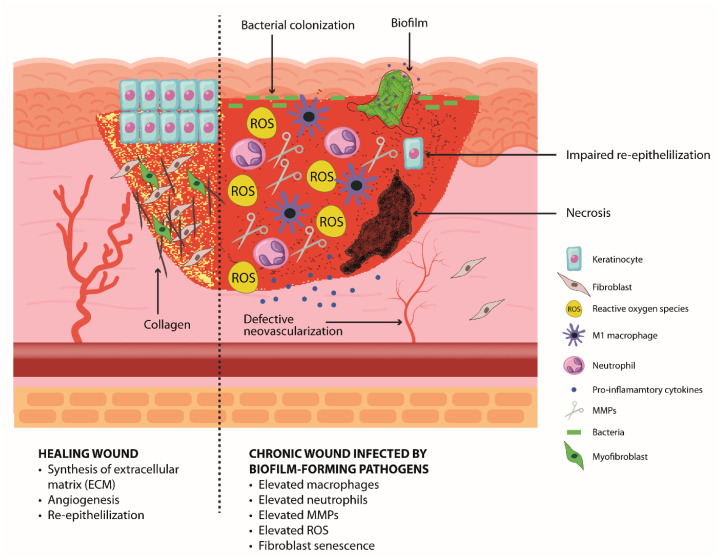
**Chronic wound.** A chronic wound is characterized by stagnation into the inflammatory phase so that excess neutrophils and macrophages are present within the wound with exaggerated ROS production and ECM degradation. This situation promotes bacteria colonization and the formation of biofilms, which further impair healing and a response to treatments. Chronic wounds are characterized by defective angiogenesis that leads to necrosis, impairment in the re-epithelialization process, and the senescence of fibroblasts that causes insufficient ECM production.

The characteristics of a chronic wound compared to those of a healing wound are depicted in Figure 2. 

## 5. Pathogen Colonization and Chronicity of Wounds

Wounds constitute a great opportunity for microorganisms belonging to the skin microbiota and to the environment to gain entry into deeper tissue and find the optimal condition for colonization and growth [52,53]. Chronic wounds are disposed to bacterial infection, leading to serious complications. Managing wounds in the presence of infection is still challenging due to prolonged healing and frequent reoccurrence [7]. The recent development of new-generation sequencing targeting the species-specific small subunit ribosomal RNA (16S rRNA) gene and shotgun metagenomics allows the characterization of the skin and wound microbiota thanks to the ability to identify anaerobic microorganisms [54]. Different wound microbiome studies found that in chronic wounds, mixed communities of Gram-positive anaerobic bacteria can inhabit the deeper tissue of the skin [54,55], and their persistence after debridement is associated with poor wound outcomes [56]. Bacteria that colonize wounds belong to 21 families, in particular Staphylococcaceae and Pseudomonaceae, regardless of the etiology of the wound [57,58,59,60]. Chronic wounds of different etiologies showed that *Staphylococcus* and *Pseudomonas* were the most common genera, 63% and 25%, respectively [61]. A study on chronic venous leg showed that Gram-negative bacteria constituted 51.7%, mostly represented by *Pseudomonas aeruginosa*, followed by *Escherichia coli*, *Serratia marcescens*, *Enterobacter cloacae*, and *Morganella morganii*, while the Gram-positive bacteria constituted 48.3%, with *Staphylococcus aureus* as the dominant species [7]. Indeed, the colonization of the chronic wound is characterized by the presence of multiple microorganisms, initially by Gram-positive belonging to *Staphylococcus* genera, which do not elicit strong immune responses [62], and subsequently, by Gram-negative bacteria such as *Pseudomonas* spp., *E. coli*, *Klebsiella pneumoniae,* and *Enterobacter* spp., which become the predominant species in the microenvironment. This colonization leads to an increase in the bioburden, causing a delay in the healing process. Microbial products released by pathogenic bacteria can trigger various pathways of cytokine production to promote their survival and persistence within the host [7]. In particular, the deregulation of anti-inflammatory cytokine IL-10 is associated with virulence strategies leading to poor antimicrobial effector mechanisms, increased disease severity, and chronicity in *S. aureus* and *P. aeruginosa* infections [63]. Recently, it has been observed that bacteria in chronic wounds develop strategies that contribute to persistent infection and the prolongation of the inflammatory stage, resulting in collateral damage to the adjacent host tissue and, consequently, a delay in healing. 

### 5.1. Biofilm in Chronic Wound Infections

#### 5.1.1. Biofilm Development and Characteristics

**Figure 3:**
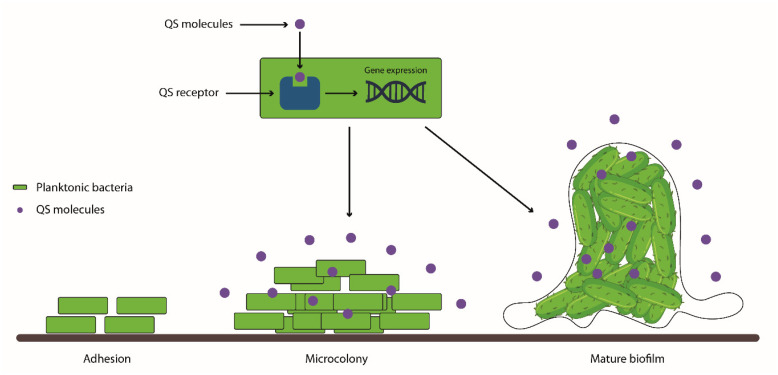
**The biofilm formation process.** Planktonic bacteria adhere to a surface or a wound and form a microcolony in which they start to deposit the extracellular polymeric substance, leading to the maturation of the biofilm. In both the microcolony and mature biofilm, the extracellular polymeric substance is produced thanks to QS signaling pathways that, by regulating gene expression, also regulate metabolism, virulence, and several other cellular functions.

The ability of pathogenic species to produce multicellular structures, called biofilms, is frequently associated with difficult-to-treat infections and high mortality rates [64]. Biofilms are defined as multicellular structures consisting of multiple bacterial species that adhere to a surface and are enclosed within an extracellular polymeric substance (EPS) [7,65]. This matrix is composed of polysaccharides, proteins, glycolipids, and extracellular DNA capable of protecting bacteria and facilitating intercommunication through chemical and physical signals, increasing both the integrity and strength of the biofilm [61,66]. Biofilm formation is regulated by quorum sensing (QS), a type of communication between bacterial cells based on chemical signals that modulate several cellular functions. The biofilm formation process occurs through the adhesion of planktonic bacteria, which is followed by microcolony formation and the maturation of the biofilm in which microorganisms behave as a community (Figure 3) [67]. Biofilms support survival in adverse environments, the evasion from the host immune system, and the resulting long-term persistence [68].

#### 5.1.2. Pathogenesis and Treatment Challenges of Chronic Wounds Infected with Biofilm-Forming Pathogens

Chronic wounds represent an ideal environment for biofilm formation as necrotic tissue and debris allow for bacterial attachment, and wounds are more susceptible to infection due to an impaired host immune response [69,70]. Both pathogenic and nosocomial bacteria have been observed to exist as biofilm producers in the natural environment as well as in infected tissues as polymicrobial communities [71]. Biofilm formation was found to be the main cause of many chronic infections, such as DFU and necrotizing fasciitis, which lead to the re-emergence of multidrug-resistant strains and result in treatment failure [72]. Although many species can form this type of structure, *P. aeruginosa* and *S. aureus* have been reported to be the most common microbes responsible for biofilm formation in chronic wounds [73]. Both types of bacteria release virulence factors, including toxins and enzymes, which promote their adherence to the damaged tissue and decrease the immune response of the host, leading to further tissue damage [74]. For example, it has been shown that rhamnolipid, a leukocidal toxin produced by *P. aeruginosa*, causes the rapid necrosis of polymorphonuclear leukocytes [75]. Besides this, the diabetic condition is associated with slower wound healing and increased biofilm thickness [70]. In addition, the DFUs showed a high bacterial complexity, making these infections complicated to manage. Several studies demonstrated synergistic interactions between *S. aureus* and *P. aeruginosa* in DFUs, which can enhance their colonization, virulence, or persistence. In particular, many substances produced by *P. aeruginosa* may play a protective role for *S. aureus* [76]. Biofilms are commonly resistant to topical and systemic antibiotics, reducing the effectiveness of the antimicrobial treatment thanks to the presence of the exopolysaccharide matrix, which acts as a mechanical barrier protecting the cells from the entry of the antibiotic and the immune system. Moreover, biofilms allow for the exchange of plasmid-mediated antimicrobial resistance genes among bacteria. Indeed, analyzing 153 strains from chronic wounds, Di Domenico and collaborators found 74.4% of multi-drug resistant organisms (MDROs) and 78.9% of non-MDROs with a comparable ability to form biofilms [77]. Increased mutation frequencies have been described in the biofilm cultures of *P. aeruginosa*, *S. aureus*, *S. epidermidis,* and *Streptococcus pneumoniae*, suggesting that biofilm matrix is a favorable environment to promote mutational resistance to antibiotics [78]. Biofilms are present in 60% of chronic wounds and in only 10% of acute wounds; however, since there are no specific clinical manifestations, their presence is probably underestimated [79]. Biofilms may be responsible for inducing chronic inflammation, and indeed, an increase of specific inflammatory mediators, such as IL-6, IL-10, IL-17A, and TNF-α, was found in wound fluids in the presence of an infection, in particular, when sustained by biofilm-producing bacteria, influencing ulcer size. The continuous stimulation of the immune system can lead to the worsening of chronic inflammation and perpetuate the cycle of the chronic wound. Moreover, biofilms contribute to wound bed senescence caused by oxidative stress and protease-mediated degradation of receptors and cytokines [80]. 

Considering all aspects of chronic wounds infected by biofilm-forming pathogens, new therapeutic approaches are necessary. Therapies that target the bacteria-innate immune interactions to ameliorate antibacterial immune responses and/or regulate inflammatory responses seem to be very promising [81]. New therapeutic approaches also aim to improve the role of commensal microorganisms in counteracting the colonization of pathogenic species. In fact, these can produce several AMPs that inhibit the growth of pathogenic strains and induce an immune response of the host against them [82]. As alternatives, the preparation of purified or engineered AMPs, bacteriophages, nanoparticles, and molecules that inhibit QS are under investigation. 

## 6. Biofilm Wound Treatment 

Upon confirmation of an infection in the chronic wound, it is essential to define the best therapeutic strategies, and managing and controlling biofilm formation are crucial aspects of wound care [83]. The gold standard in the management of chronic wound biofilms consists of systemic antimicrobial therapy in combination with the debridement of wounds [84]. In particular, wound debridement is a fundamental step in biofilm management, as it removes devitalized tissue, debris, and the outer layers of the biofilm, and thus helps expose the underlying microbial populations to other treatment modalities [85]. This process can allow wounds to progress beyond the inflammatory stage towards healing as it prepares the wound bed for re-epithelialization [86]. This can be accomplished by various methods, such as sharp, mechanical, autolytic, and enzymatic debridement [87]. Sharp debridement involves the use of surgical instruments, such as scalpels, to precisely remove necrotic tissue and biofilm from the wound [85]. The limitations of debridement are the impossibility of eliminating all the microbes and obtaining clear margins, and the impossibility of the management of some patients through conservative or surgical debridement due to comorbidity-associated contraindications. Autolytic debridement is the natural process by which endogenous phagocytic cells and proteolytic enzymes break down necrotic tissue [88]. Enzymatic debridement requires the activity of proteolytic enzymatic agents, such as collagenase and papain, to dismantle necrotic tissue [89]. Indeed, clostridial collagenase exhibits activity in the pH range found in most chronic wounds and achieves selective debridement by digesting denatured collagen in eschar while sparing non-necrotic tissues [90]. Although enzymatic debridement is an easy, feasible, and highly selective process whereby only necrotic tissue will be affected in the debridement, it requires time to achieve successful results [91], and it is usually used in combination with surgical debridement [92]. Other methods, like wet-to-dry dressings or hydrotherapy, use mechanical force to remove dead tissue and biofilm. While they are less selective than sharp debridement, as they remove devitalized tissue, debris, and viable tissue, they can be effective in certain cases [88]. Furthermore, wound debridement induces a notable “microbiome shift” in the wound’s microbial community, resulting in a decrease in low-virulence pathogenic anaerobes, which ultimately leads to a more favorable outcome [93]. Kalan and collaborators reported a significant reduction in Shannon diversity that was not dependent on a change in the relative abundances of aerobic bacteria such as *S. aureus*, *Streptococcus agalactiae*, and *P. aeruginosa*; however, they observed a reduction in mixed anaerobic bacteria, such as *Anaerococcus lactolyticus*, *Porphyromonas somerae*, *Prevotella melaninogenica*, and *Veillonella dispar* after debridement in healed wounds but not in unhealed wounds [73]. Unfortunately, due to the difficulty of completely removing biofilms through surgical wound debridement, these strategies showed limited success [94]. Even if the use of antibiotics seems to be crucial for the treatment of chronic wounds, it could drive antibiotic resistance and lead to adverse advents. Indeed, the use of systemic or topical antibiotics for treating wound infections is not recommended due to limited evidence of their effectiveness and frequent selection for resistant colonizing bacteria [95]. In order to achieve better outcomes, several new treatment strategies have recently been developed to destroy biofilms. Among novel strategies of note are the nonionic surfactant gel utilizing a poloxamer polymer (Plurogel™) [96], the sustained-release iodine dressing (Ioplex™, Iodosorb™, Iodoflex™) [97,98,99] and negative pressure wound therapy (NPWT) [100]. These new strategies have been reported to reduce the total bacterial bioburden of infected wounds. 

The emerging strategies aim to promote wound healing by restoring the healthy skin microbiota either by inducing the colonization of the wound by beneficial microorganisms or by interfering with the signaling pathways responsible for the virulence of pathogenic species. These strategies take advantage of topical probiotics [101,102] or natural or synthetic molecules that inhibit QS and thus interfere with the biofilm formation process [103]. Interestingly, it has been reported that probiotics accelerate chronic wound healing by competing with pathogenic species and thus reducing bacterial load [104]. Moreover, probiotics interfere with the QS of pathogenic species and, therefore, are able to either destroy biofilms or prevent their formation [105]. Recently, *Lactobacillus rhamnosus* was demonstrated to be effective against *Acinetobacter baumanii* biofilms in in vitro experiments. Therefore, its use in chronic infections caused by this pathogen has been suggested. The authors showed that the observed effect was mainly exerted by lactic acid and acetic acid, which showed a strong anti-*A. baumanii* bactericidal effect [106]. In another study, the authors demonstrated that the *Lactobacillus plantarum* strain was able to interfere with *P. aeruginosa infections* by suppressing QS, adhesion, and biofilm formation. In addition, they also showed that *Lactobacillus acidophilus*-derived substances are able to disrupt *S. aureus* and *S. epidermidis* biofilms by influencing cell-to-cell and cell-to-surface interactions [107]. These results were confirmed by another group that efficiently inhibits *P. aeruginosa* biofilm formation in vitro using *L. acidophilus* preparations or their cell-free filtrates. In this work, the authors showed that high concentrations of cell-free filtrates were also able to destroy pre-existing *P. aeruginosa* biofilms. Therefore, the authors demonstrated that the concentration of substances released by *L. acidophilus* determines whether the effect will be of biofilm formation inhibition or also in dismantling established biofilms [108]. Of great interest is the use of the *L. plantarum* strain in chronic wounds, which has been first described in in vitro and in vivo assays, and afterward, their usefulness in promoting wound repair in clinics has also been described. The authors showed that *L. plantarum* is able to antagonize *P. aeruginosa* in vitro and in an in vivo mouse model, inhibiting the synthesis of QS molecules and thus inhibiting adhesion, biofilm formation, and the expression of virulence factors, and increasing phagocytic activity [104]. In addition, the *Lactobacillus fermentum* RC-14 strain was shown to efficiently inhibit surgical implant colonization by *S. aureus*, the formation of implant biofilms in a rat model [109], and to have anti-biofilm activities against several pathogens in vitro [110]. Afterward, the treatment of chronic wounds with the topical application of 10^5^ *L. plantarum* was used in the clinical setting in the treatment of burn wounds. In this work, the wounds were treated once a day for 10 days, exclusively with a preparation of *L. plantarum* or with silver sulphadiazine. This microbicidal agent represents the standard of care in burn wounds. Authors suggested that treatment with *L. plantarum* was as effective as the standard of care in preventing infection, promoting granulation tissue and healing. Notwithstanding, in this work authors admit that the low number of patients prevented from applying statistical power [111]. In another work, physicians successfully treated a case of multidrug-resistant and multi-strain chronic wound infection (*Klebsiella pneumoniae*, *E. faecalis*, and *Proteus mirabilis*) with topical probiotics. A lyophilized preparation of probiotics containing 100 billion colony-forming units (CFUs) of *L. plantarum* NCIBMB 43029 20% in weight, *L. acidophilus* NCIBMB 43030 20% in weight, and *Streptococcus thermophilus* NCIMB 30438 40% in weight was applied to the wound for 24 days. In this case, previous treatment with antibiotics failed, and complete wound healing was obtained in 90 days, treating the wound exclusively with the topical application of this preparation of probiotics of the *L. plantarum*, *S. thermophilus*, and *Lactobacillus acidofilus* strains [112]. Although very promising, the use of this particular preparation of probiotics has been used only in one clinical case, and further studies are needed to verify the efficacy of this therapy in a larger population. In addition, the concentration of probiotics used is not easily reproducible since a proportionally reduced amount of the lyophilized preparation was used to cover the contracting wound [112]. In this scenario, using a specific concentration of probiotics per mm^2^ of wound seems to be a valuable strategy to increase reproducibility. Recently, the use of topical probiotics in chronic wounds by taking advantage of delivery strategies, such as microencapsulation, hydrogels, microneedling, and electrospun scaffolding, has been proposed [101]. Through these strategies, it would be possible to maximize the effect of probiotics on wound healing.

As an alternative, a strategy based on the use of anti-QS natural or synthetic molecules able to inhibit biofilm formation and promote their degradation could be used [113]. The use of QS inhibitors seems to be particularly promising since they are able to hamper the virulence of *S. aureus and P. aeruginosa*, inhibiting their growth [114]. In particular, lactonases were shown to be very promising, since they inhibit the QS of *P. aeruginosa* and thus interfere with the virulence of this pathogenic species. Among lactonases, the engineered molecule SsoPox-W263-I was shown to be effective in reducing in vitro the expression of virulence factors and biofilm formation of *P. aeruginosa* strains isolated from DFUs by disrupting QS [114]. The anti-virulence potential was confirmed in an amoeba infection model in which SsoPox-W263-I was able to efficiently decrease the virulence and biofilm formation of *P. aeruginosa* strains isolated from DFUs [115]. Even though the results are promising, considering the high thermal stability of the molecule, to date, no studies have been performed in humans. Besides this, the important role of curcumin and luteolin as natural QS inhibitors has been described. Curcumin has been shown to repress the expression of QS genes, decrease virulence, and suppress the biofilm formation of *Bacillus subtilis* and *P. aeruginosa* [116]. However, since the use of curcumin is hampered by its toxicity at high concentrations, low availability, and instability, curcumin nanoformulations have been developed. Nanoparticles (NPs) can cross biological barriers and, therefore, can efficiently release the antimicrobial agent directly within biofilms [117,118]. Indeed, the curcumin-loaded chitosan tripolyphosphate NPs proved to be effective in improving the pharmacokinetics of curcumin in vitro and in animal models [119,120]. Similar to curcumin, luteolin was shown in in vitro and molecular docking analysis to be able to inhibit *P. aeruginosa* motility, virulence, and biofilm formation by attenuating the accumulation of QS molecules and downregulating QS signaling molecules [121]. Moreover, it has also been shown that luteolin inhibits the biofilm formation and cytotoxicity of *S. aureus* in vitro by blocking bacterial toxin synthesis [122]. Nonetheless, the possible efficacy of luteolin in treating patients with chronic wound infection is not known. Besides being used to deliver active molecules in chronic wound settings, NPs can have antimicrobial activity themselves. For example, silver NPs display an intrinsic antimicrobial activity that, alongside their antibiofilm actions, makes them suitable to increase the vulnerability of bacteria to antibiotics or other antibacterial molecules [123]. Another interesting strategy to reduce *P. aeruginosa* biofilms relies on the activity of bacteriophages. Bacteriophages were shown to be useful either in inhibiting *P. aeruginosa* biofilm formation by stopping QS or destroying the biofilm structure [124]. Bacteriophages have been successfully used in patients with chronic wounds that failed to respond to local debridement and antibiotic therapy. In this study, 20 patients were enrolled and treated topically with a cocktail of customized bacteriophages. All patients showed signs of healing with the formation of granulation tissue, and seven patients achieved complete healing after three weeks of treatment [125]. Bacteriophage therapy for the treatment of *P. aeruginosa* wound infections in burned patients was also tested. In this study, 27 patients were treated with a cocktail of 12 natural lytic *P. aeruginosa* bacteriophages (PP1131 at a concentration of 1 × 10^6^ plaque-forming units (PFU) per mL) or with the standard of care (1% sulfadiazine silver emulsion cream). Both treatments resulted in a successful reduction in bacterial burden. However, bacteriophage therapy took a longer time to work. The authors suggested that further studies using higher bacteriophage titers and a larger population are needed to describe the outcomes of this treatment better [126].

Other strategies, such as passive or active immunotherapies that promote the killing of biofilm-forming species, are also valuable potential therapeutic tools. Interestingly, Ramezanalizadeh et al. tested an active immunotherapy that consisted of a vaccine in which a combination of antigens from both the planktonic and the biofilm forms of *A. baumanii* was used. This combined vaccine was able to elicit high IgG antibody titers that led to complete bacterial clearance [127]. Since a previous work of the same group showed only partial protection when using vaccines based only on the antigens of planktonic or biofilm forms [128], in this work, the authors suggest that in order to elicit an efficient immune response, vaccines should consist of the antigens of both the planktonic and biofilm forms [127]. On the other hand, passive immunotherapy using avian IgY immunoglobulins targeting *P. aeruginosa* represents an alternative to conventional antibiotic therapeutics. Indeed, Ahmadi et al. showed that anti-flagellin IgY antibodies were able to protect murine model burn wounds against *P. aeruginosa* infection [129]. In addition, the same group showed that in a murine model of burn wounds, polyclonal anti-whole cell IgY against *P. aeruginosa* was effective in reducing the bacterial load by interfering with virulence factors, motility, and biofilm formation [130]. Furthermore, several antibodies that target biofilm components were shown to be effective in pre-clinical studies, but none of them proved to be effective in clinical studies [131].

Another passive immunotherapy could consist of the use of AMPs. AMPs are amphipathic oligopeptides that display antimicrobial activity through their interaction with the phospholipids of microbial membranes, causing the formation of pores and subsequent cell lysis [132]. They are very promising antimicrobial molecules since they are able to effectively kill bacteria and, due to very low chances of the development of resistance, they represent valid alternatives to antibiotics [85]. AMPs are also able to target biofilm-specific features, probably acting as QS inhibitors or down-regulators of extracellular matrix biosynthesis. AMPs also recruit the host immune cells at the site of infection and modulate the inflammatory response [133]. Of interest, it has been demonstrated that several AMPs promote the re-epithelisation and granulation of tissue and, in this way, support wound healing. Indeed, the endogenous antimicrobial host defense protein S100A8/A9 has been shown to be absent in chronic wounds and to be an important host defense mediator in DFU [134,135]. In addition, a broad-spectrum engineered cationic antibacterial peptide PLG0206 (WLBU2) was shown to inhibit *P. aeruginosa* and *S. aureus* biofilms. This peptide is currently under investigation in a phase I trial that aims to verify its ability to treat *P. aeruginosa* infections of the prosthetic joints [136]. Notwithstanding, the low solubility, instability, and low availability, when used topically, could limit the use of AMPs at the clinical level [85]. To enhance wound repair mechanisms, nanocarriers could be used as effective AMP delivery systems. Indeed, nanocarriers can offer the advantage of protecting AMPs from degradation, improving their pharmacokinetic profile, and reducing toxicity. Moreover, nanocarriers themselves can also have beneficial effects on different stages of wound healing. Several NPs, among which are poly(lactic-co-glycolic acid) (PLGA) NPs, chitosan NPs, and lipid carriers, have been developed to enclose AMPs with the purpose of promoting wound healing. Besides delivering AMPs, PLGA NPs serve as a lactate supply, which has been associated with accelerated angiogenesis and recruitment of endothelial progenitor cells and enhanced wound healing process [137]. Also, the incorporation of potent cationic antimicrobial polymers into neutral hydrogels that protect the wound and conditions advantageous to wound healing showed to be a promising and clinically translatable strategy to treat resilient wound biofilm infections [138]. 

**Figure 4:**
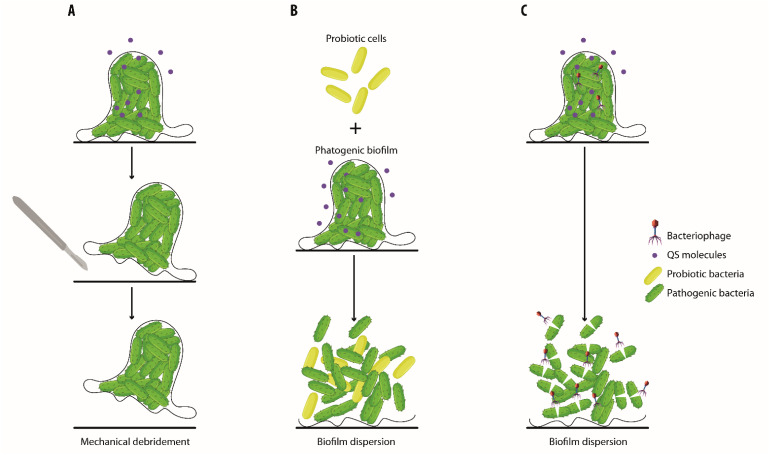
**Strategies to reduce biofilms and to promote chronic wound healing.** Biofilms can be displaced by mechanical debridement (**A**) or dismantled either by the use of probiotics (**B**) or bacteriophages that lead to the lysis of pathogenic bacteria (**C**).

**Figure 5:**
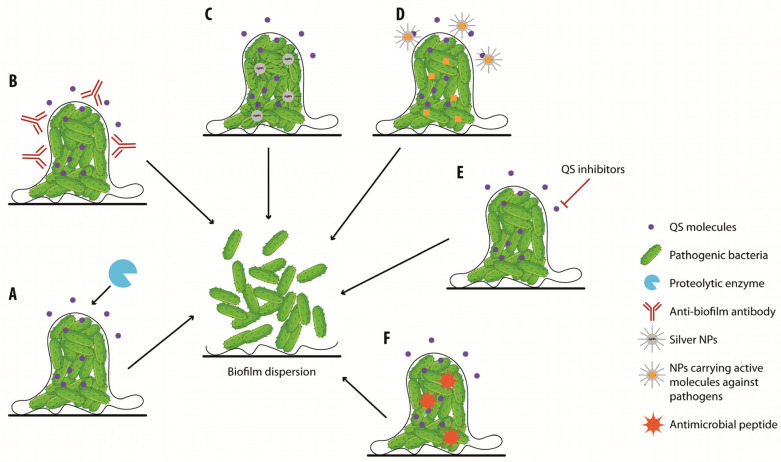
**Strategies to reduce biofilms and to promote chronic wound healing.** Biofilms can be dismantled either by enzymatic debridement (**A**), anti-biofilm antibodies (**B**), nanoparticles (**C**), nanoparticles for anti-biofilm agent (orange rectangles) delivery (**D**), QS inhibitors (**E**), or by the use of antimicrobial peptides (**F**).

Figure 4 and Figure 5 depict the strategies to reduce the formation of biofilms or to dismantle pre-existing biofilms.

In this context, it is worth noting that the combination of these strategies has the potential to promote faster wound healing and decrease the frequency of serious outcomes [139].

## 7. Conclusions and Future Directions

Chronic infected wounds represent a major public health concern that leads to frequent hospitalization and even death. Efforts have been made to define the role of the microbiome in the wound-healing process and the mechanisms used by microorganisms to chronically colonize wounds by forming biofilms. In this context, the restoration of the normal skin microbiota by preventing or eliminating pathogen colonization is essential to promote wound healing. In this context, treatments that inhibit biofilm formation or are able to destroy pre-existing biofilms are essential. Recently, new strategies for wound debridement, the use of topical probiotics, natural or synthetic anti-QS molecules, bacteriophages, and NPs, or the use of passive or active immunotherapies demonstrated promise for the treatment of chronically infected wounds in the near future. Importantly, the use of combinations of these approaches alongside strategies to deliver active molecules into biofilms, based on nanoformulations, could lead to efficient pathogen killing, biofilm destruction, and finally, wound healing. Also, the study of possible combination strategies that target cells in different metabolic states or environmental niches is essential to be effective against microbial biofilms. Indeed, the combination of different antibodies, molecules, or NPs able to target both the planktonic and biofilm bacterial forms seems to be very auspicious. Besides this, strategies that combine the use of antibiotics or debridement strategies with the use of AMPs, QS inhibitors, NPs or bacteriophages could potentiate the effect of the treatments alone. It should also be considered that the treatment of chronic wounds with topical probiotics could synergize with passive or active immunotherapies or QS inhibitors. 

To conclude, even though the scenario of possible future anti-biofilm strategies appears to be outstanding, large and controlled studies are needed to confirm the efficacy and safety of these novel approaches and their combination to make them translatable into the clinical setting. 

## Data Availability

Not applicable.
